# Sensor-Based and Visual Behavioral Profiling of Dry Holstein Cows Presenting Distinct Median Core Body Temperatures

**DOI:** 10.3390/ani14192832

**Published:** 2024-10-01

**Authors:** Nicolle F. F. Bönmann, Luis G. D. Mendonça, Isabella Sellmer Ramos, Rebecca Fritz, Caio Gamarra, Douglas Duhatschek, Raphael S. S. de Oliveira, Alexandre L. A. Scanavez, Thiago S. Belem, Matthew C. Lucy, Joao G. N. Moraes

**Affiliations:** 1 Department of Animal and Food Sciences, Oklahoma State University, Stillwater, OK 74078, USA; niferri@okstate.edu (N.F.F.B.);; 2 Department of Animal Sciences and Industry, Kansas State University, Manhattan, KS 66506, USAalex.scanavez@altagenetics.com (A.L.A.S.); 3 Division of Animal Sciences, University of Missouri, Columbia, MO 65211, USA

**Keywords:** heat stress, accelerometer, behavior, activity, transition period

## Abstract

**Simple Summary:**

Heat stress results in significant economic losses to the dairy industry. At the individual cow level, core body temperature (CBT) serves as a reliable proxy for quantifying the degree of heat stress experienced by cows. As animals adapt their behavior to cope with heat stress, this study investigated behavioral patterns in dry Holstein cows with distinct CBT during the summer months, a season known for inducing heat stress in dairy cattle. Behavioral patterns were measured through visual observations and by automated activity monitoring capturing patterns such as lying, standing, eating, activity level, and rumination. Using sensor technology tracking activity 24/7, we observed that cows classified as high-temperature (HT) exhibited higher periods of heightened activity and lower periods of inactivity prepartum and diminished rumination time postpartum than low-temperature (LT) cows. Since rumination time is a key indicator of dairy cow health, our results suggest that HT cows faced greater health challenges postpartum. We also identified many differently expressed genes (DEGs) previously associated with heat stress in immune cells from HT vs. LT cows. Collectively, our results suggest that the observed behavioral and physiological differences between HT and LT cows are linked to their distinct tolerance to heat stress.

**Abstract:**

The consequences of heat stress during the dry period can extend into the postpartum period, affecting health and productivity in the subsequent lactation. We hypothesized that cows with distinct core body temperatures (CBTs) would exhibit disparate behaviors associated with different degrees of heat generation or dissipation. The primary objective was to investigate behavioral differences of dry Holstein cows (*n* = 50) classified as high-temperature (HT) or low-temperature (LT), based on median CBT during the summer months using visual observations and accelerometer technology. A secondary objective was to investigate the transcriptome of white blood cells (WBCs) collected from a subgroup of HT and LT cows (*n* = 5; per group). Minor behavior differences were observed during the visual observations (performed for a total of 16h/cow). Based on automated monitoring system (AMS) data, collected 24/7 over a period of 42 days per cow, HT cows displayed higher periods of high activity and lower periods of inactivity prepartum and diminished rumination time postpartum than LT cows. There were 16 differently expressed genes (DEGs) in WBCs of HT compared to LT cows. Several of the identified DEGs have been previously associated with heat stress. The observed trends in the AMS data indicate that CBT and patterns of activity prepartum may serve as valuable predictors for identifying dairy cows with distinct tolerance to heat stress.

## 1. Introduction

Economic losses due to heat stress are estimated to range between USD 0.9 and USD 1.5 billion annually for the dairy industry [1]. Successful dairy enterprises, therefore, prioritize cow comfort to mitigate the adverse impacts of elevated temperatures [2]. Heat stress in dairy cattle begins at a relatively low temperature–humidity index (THI) of 68 [3,4,5]. Adverse impacts on milk yield and reproductive efficiency were observed when THI reached or exceeded 69 [3]. Various research groups have further corroborated the fact that heat stress adversely affects milk production, postpartum health, and reproductive efficiency in dairy cows [6,7,8,9,10].

At the individual cow level, core body temperature (CBT) serves as a reliable proxy for quantifying the degree of heat stress experienced by cows. Karimi et al. [11] reported that small differences in rectal temperature of dry cows under heat stress or subjected to cooling (39.5 °C vs. 39.2 °C, respectively) during the close-up period were associated with greater respiration rates, decreased dry matter intake (DMI), decreased rumination, decreased lying duration, and decreased milk yield during the first 180 days in milk (DIM). Additional studies, including ours [9,12], which categorized cows into high-temperature (HT) and low-temperature (LT) groups based on median CBT during the dry period, support the notion that even modest variations in average CBT (~0.3 °C) due to heat stress are associated with the development of postpartum diseases and reduced milk production in the subsequent lactation. Because animals adapt their behavior to cope with heat stress [13], it is conceivable that HT and LT cows may exhibit divergent behaviors influencing heat generation or dissipation. Such behavioral disparities could result in differing degrees of tolerance or susceptibility to heat stress, subsequently impacting health and productive outcomes postpartum.

Behavioral tendencies can be measured through visual observations or by automated activity monitor systems that capture patterns such as lying, standing, eating, activity level, and rumination. Several groups have demonstrated a relationship between the health status of transition cows and behavior captured by sensor technologies [14,15,16,17,18,19,20]. The amount of time cows spend ruminating, for instance, is an important indicator of postpartum health and is negatively correlated with THI [21]. Besides influencing rumination, heat stress is associated with additional behavioral and physiological changes in dairy cows, including alterations in the duration of lying bouts [22,23], eating behavior and DMI (West, 2003 [24]), and water consumption, to cope with hyperthermia [25]. Therefore, the current study hypothesized that cows classified as HT and LT based on median CBT during late gestation [9,12] would exhibit distinct behavioral patterns during the pre and postpartum periods. Hence, the primary objective of this study was to investigate behavioral differences of dry Holstein cows classified as HT or LT during the summer months using a dual approach that incorporated visual observations and accelerometer technology. A secondary objective was to investigate the transcriptome of white blood cells (WBCs) from a subgroup of HT and LT cows. Thermal stress initiates a range of adaptive physiological and cellular responses, including the induction of heat-shock proteins (HSPs) that alleviate hyperthermia and mitigate the risk of mortality derived from extended cellular damage [26]. Given that circulating WBCs have been observed to react to heat stress in humans, even prior to any notable rise in core temperature following heat exposure [27], the transcriptome analysis of WBCs was intended to provide insights into the genetic and molecular mechanisms that underlie the physiological differences in temperature regulation between HT and LT cows, rendering them more susceptible or resilient to heat.

## 2. Materials and Methods

### 2.1. Animals, Facilities, Management, and Enrollment

**Figure 1:**
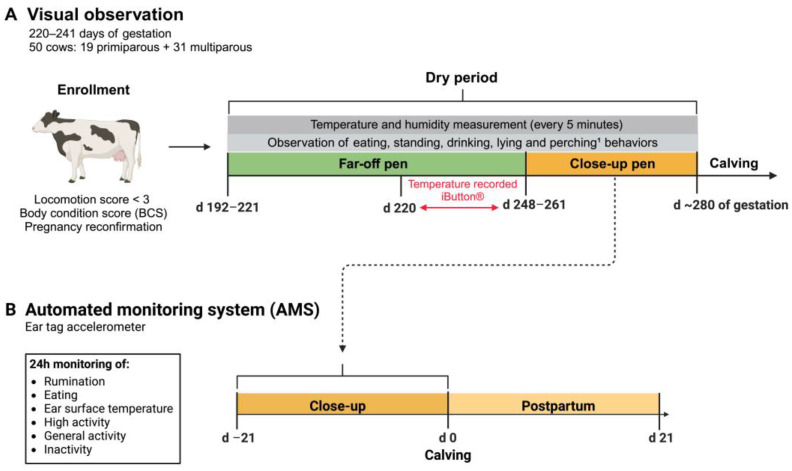
Schematic figure of the experimental study design. (**A**) 50 animals (between 220 and 241 days of gestation and with locomotion score < 3) were enrolled in the study. Lactating Holstein cows were dried off between 192 and 221 days of gestation and moved to the far-off dry pen. Core body temperature (CBT) was recorded for 7 days using a temperature logger (iButton) placed intravaginally in cows between day 220 and 241 of gestation. Cows were moved to the close-up pen between 248 and 261 days of gestation. Each cow was observed visually for 16 h, including 8 h in the far-off pen and 8 h in the close-up pen. Each 8 h observation block consisted of two 2 h morning observations (0600 to 0800 h) and two 2 h afternoon observations (1600 to 1800 h). During the visual observations, five behaviors were recorded: eating, standing, drinking, lying, and perching. ^1^ Perching was only recorded in the far-off pen because the close-up pen did not have free stalls. (**B**) Representative timeline for the automated monitoring system (AMS) employed for tracking. Accelerometer data on rumination, eating, activity (e.g., high activity, general activity, inactivity), and ear surface temperature were collected continuously (24 h/day, on a minute-by-minute basis) from day –21 to day 21 relative to calving (day 0), and analyzed.

The study was conducted at the Kansas State University Dairy Teaching and Research Center from June through November 2019. Lactating Holstein cows were dried off between 192 and 221 d of gestation and were moved to the far-off dry pen (Figure 1). Once dry for at least 6 d, cows at 220 to 241 d of gestation having a locomotion score < 3 (1 = not lame and 3 = noticeably lame; [28] were enrolled in the study. A total of 50 cows were enrolled (19 primiparous and 31 multiparous [19 s-lactation cows and 12 third-lactation or greater]) in five replicates. Cows were reconfirmed pregnant at the time of enrollment. Body condition score was evaluated on the day of enrollment (d0), and again at 7 and 14 d after enrollment, using a 5-point scale with 0.25-point increments [29].

Cows were moved to the close-up pen between 248 and 261 d of gestation. Cows in the far-off pen were housed in a free-stall barn and fed a total mixed ration once daily, with free access to prairie hay and water. No active cooling was provided in the far-off pen; however, shade was provided over free-stall beds and feed bunks. Cows in the close-up pen were housed in an open-front barn on straw bedding over concrete, provided with a total mixed ration once daily, and had free access to water. The back wall of the barn was equipped with cellulose cooling pads and fans. After calving, lactating cows were housed in free-stall barns equipped with sprinklers and fans and fed a TMR once daily (twice daily during summer). Lactating cows are milked thrice daily, starting at 0400 and 1600 h.

### 2.2. Temperature Humidity Index (THI) and Assessment of Core Body Temperature (CBT)

**Figure 2:**
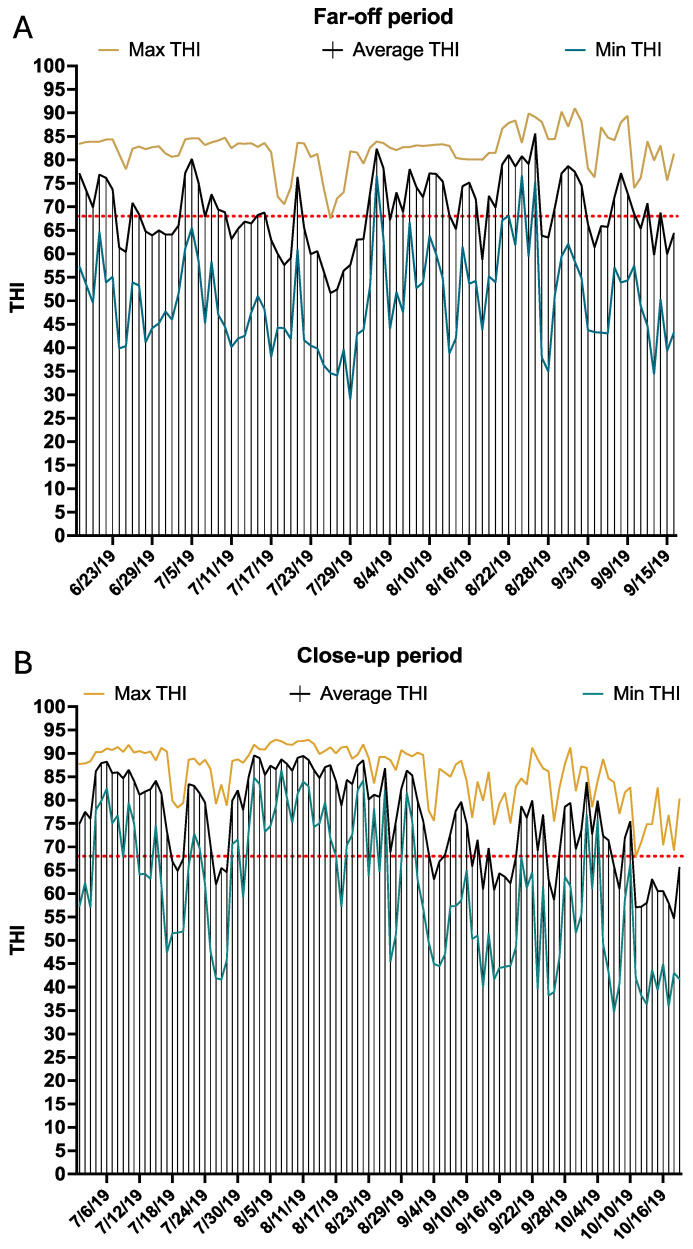
Ambient temperature and humidity were monitored in both the far-off (**A**) and close-up (**B**) pens by fixing a temperature logger (HOBO U23 Pro v2, Onset Computer Corp., Pocasset, MA, USA) in each pen. Temperature and humidity measurements were recorded every 5 min in both pens. Temperature data were downloaded from the loggers and used to calculate THI. The daily maximum (yellow line), average (black line), and minimum (blue line) THI values are represented for the days when the cows enrolled in the study were in the far-off pens (**A**) and close-up pens (**B**).

Ambient temperature and humidity were monitored in both the far-off (Figure 2A) and close-up (Figure 2B) pens by fixing a temperature logger (HOBO U23 Pro v2, Onset Computer Corp., Pocasset, MA, USA) in each pen. Loggers were located approximately 3 m above the ground. Temperature and humidity measurements were recorded every 5 min in both pens. Temperature data were downloaded from the loggers and used to calculate THI, as described in [9].

Core body temperature (CBT) was recorded by attaching a temperature logger (iButton DS1922L, Embedded Data Systems, Lawrenceburg, KY, USA) to a blank intravaginal insert (CIDR, Zoetis, Parsippany, NJ, USA). The iButton was attached to the insert with silicone aquarium sealant (Loctite^®^, Henkel Corporation, Rocky Hill, CT, USA) and wrapped with Parafilm (Parafilm^®^, Carolina Biological Supply Company, Burlington, NC, USA) once the sealant was dry. The insert was then placed intravaginally while cows were in the far-off pens and removed after 7 d when cows were moved to the close-up pen (Figure 1). The temperature logger recorded CBT every 5 min. Temperature loggers were calibrated for an accuracy of ±0.13 °C. Temperature loggers were inserted into the vagina of cows enrolled in the study between d 220 and 241 of gestation and removed between 228 and 248 days of gestation.

**Figure 3:**
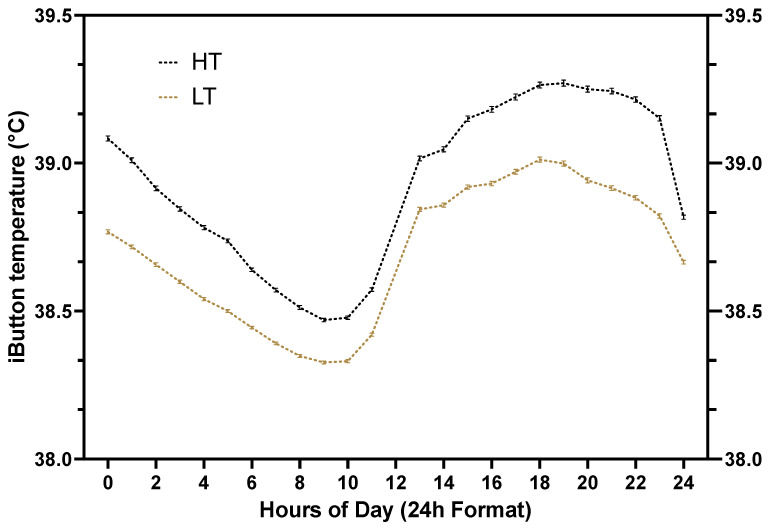
Average (±SEM) core body temperature (CBT) according to hour of the day (24 h format) for high-temperature (HT) and low-temperature (LT) cows. Core body temperature was recorded by attaching a temperature logger (iButton) to a blank intravaginal insert (CIDR). The insert remained intravaginally for 7 d and vaginal temperature was recorded every 5 min for each cow enrolled in the study. Temperature loggers were placed on study cows between d 225 and 239 of gestation and removed between 232 and 248 days of gestation. Data from 1996 ± 0.7 (mean ± SEM) temperature measurements per cow are summarized. HT, the black dotted line, represents cows with a vaginal temperature above the median value within the replicate. LT, the gold dotted line, represents cows with vaginal temperature below the median value within the replicate.

Upon insert removal, data from the loggers were downloaded to a computer and the average CBT was calculated as described by Scanavez et al., (2017 [12]). A total of 1996 ± 0.7 (mean ± SEM) temperature observations per cow were used for CBT classification (assignment to HT or HL groups). Median values were calculated for each of the five replicates. Within the replicate, 2 CBT groups were created: cows whose average CBT exceeded the median value were classified as high core-body temperature (HT), and cows with an average CBT below the median value were classified as low core-body temperature (LT). A summary of average (±SEM) CBT, according to hour of the day (24 h format), for HT and low LT cows is presented in Figure 3.

### 2.3. Assessment of Dry-Cow Behavior

#### Visual Observations

Visual observations were conducted based on the methodology described by Borchers et al. (2016) [30]. To assess behavior, all cows in the study were observed for a total of 16 h during the dry period, with 8 h allocated to the far-off pen and 8 h to the close-up pen. Seven observers, trained in continuous behavior observation, conducted the visual observations. To ensure accuracy and consistency, each observer was accompanied by another during their initial observation period. Observers were blinded to the cows’ HT or LT status, and individual cows within each replicate were randomly assigned to an observer using the Microsoft Excel ‘rand’ function. In the far-off pen, visual observations were conducted while the temperature logger collected CBT data (every other day, for a total of 4 days). In the close-up pen, visual observations were performed during the first week after moving to the close-up pen (every other day, for a total of 4 days). Each 8 h observation block comprised two 2 h morning sessions (0600 to 0800 h) and two 2 h afternoon sessions (1600 to 1800 h). These sessions were randomly scheduled over a period of 4 days, ensuring that each cow was visually observed for a total of 2 h per day across the 4-day period. Each observer was randomly assigned two cows to monitor for each 2 h block. The five behaviors recorded were: lying, standing, eating, drinking, and perching (standing with only front feet in the free stall). Perching behavior was exclusively observed in the far-off pen, as the close-up pen lacked free stalls.

### 2.4. Automated Behavior Monitoring

Automated monitoring system (AMS) data were collected using CowManager Sensor ear tags affixed to the left ear of each cow (Agis Automatisering BV, Harmelen, The Netherlands). The data captured by the ear tag equipped with accelerometers included (1) ear surface temperature, and daily minutes of (2) high activity, (3) general activity, (4) inactivity, (5) eating behavior, and (6) rumination.

The CowManager ear tag sensors capture behavior data on a minute-by-minute basis, recording the predominant behavior observed during each minute. Ear and jaw movement aid in the classification of rumination and eating behaviors, and all other behaviors that are not eating, rumination, or resting (inactivity) are classified as active. High activity is used as an indicator of estrus-like activity. Accelerometer data from d −21 to 21 relative to calving (d 0) were analyzed. Ear surface temperature was collected every hour by the ear tags. Ear surface temperature was also analyzed from d −21 to 21 relative to calving (d 0 being calving). Importantly, in bunk-fed dairy cows equipped with the sensor ear tags, feeding, and ruminating activities are significantly correlated with visual observations (simple and concordance correlations exceeding 0.82 and 0.59, respectively [30].

### 2.5. Isolation of White Blood Cells for Transcriptome Analysis

Upon removal of the blank CIDR insert housing the temperature logger from the vagina (the iButton recorded CBT data for 7 days), a blood sample was obtained from the median coccygeal vein or artery. The sample was collected into evacuated tubes containing K3 EDTA (Becton Dickinson Vacutainer Systems, Franklin Lakes, NJ, USA) for the isolation of WBC. Circulating WBCs respond to heat stress [27], and therefore differences in gene expression of WBC collected from cows in the two distinct CBT groups (HT and LT) could help elucidate physiological differences in temperature regulation between HT and LT cows, rendering them more susceptible or resilient to heat. To evaluate the transcriptome of WBCs while accounting for the effects of parity and temperature changes across replicates, blood samples collected from a subgroup of 5 HT and 5 LT multiparous cows were submitted to sequencing. Within each replicate, one HT and one LT cow were selected for transcriptome analysis, with the chosen cows exhibiting the greatest temperature differences within that replicate. Blood samples were collected between 6:00 and 8:00 a.m., and processed as described by Ortega et al. [31]. Briefly, blood samples were centrifuged at 1200× *g* for 20 min at 4 °C, and the buffy coat was transferred to a 15 mL centrifuge tube containing 12 mL of red blood cell lysis buffer (150 mM NH_4_Cl, 10 mM NaHCO_3_, 1 mM EDTA, pH 7). Tubes were vortexed and incubated at room temperature for 5 min, and then centrifuged at 300× *g* for 10 min at 4 °C to wash and pellet the WBC. After discarding the supernatant, the WBC pellet was washed twice (as described above), first using 5 mL of red blood cell lysis buffer, and second using 5 mL of ice-cold 1× Dulbecco Phosphate-Buffered Saline (DPBS). Immediately after, the supernatant was discarded, and the WBC pellet was resuspended in 1.5 mL of TRIzol™ Reagent (Invitrogen, CA, USA) and stored at −80 °C.

### 2.6. Library Preparation and Sequencing—Assessment of WBC Transcriptome

Total RNA from WBC was extracted using the TRIzol™ Reagent (Invitrogen, CA, USA) following the manufacturer’s protocol. RNA concentrations and quality were assessed using a Fragment Analyzer instrument (DNF-472, Advanced Analytical Technologies, Ames, IA, USA). Total RNA samples were sent to BGI Genomics (Beijing, China) for library preparation and sequencing. PolyA-enriched RNA libraries were constructed using an MGIEasy RNA Library Prep Set (BGI, Beijing, China) according to the manufacturer’s protocol. Briefly, mRNA was purified using oligo (dT) attached magnetic beads and then fragmented into small pieces. Following first- and second-strand cDNA synthesis, double-stranded cDNA fragments were subjected to end-repair, adenylation, adaptor ligation, and PCR amplification. Each purified library was assessed using the Fragment Analyzer, quantified with a fluorometer (Qubit; (Thermo Fisher Scientific, Waltham, MA, USA), and sequenced on an DNBSEQ G400 instrument (MGI; Shenzhen, Guangdong, China), resulting in paired-end reads of 150 bp and an average read count of 67 million paired reads per sample.

### 2.7. Statistical Analysis

All statistical analyses of behavior data captured by either the accelerometer or visual observations were conducted using SAS Studio 3.81 Enterprise Edition (SAS Institute Inc., Cary, NC, USA). All continuous data were assessed for normality using the UNIVARIATE procedure. Continuous data not normally distributed were log-transformed.

The proportions of multiparous cows (Table 1) and cows delivering twins within the CBT groups were analyzed by Chi-Square using the FREQ procedure. The remaining descriptive data were analyzed by fitting mixed-effect models using the GLIMMIX procedure. These models included CBT (HT vs. LT) as the fixed effect, with the random effect of replicate nested within CBT. Out of the initial 50 cows enrolled in the study, two cows were removed because of lameness and spontaneous late-term abortion during the far-off period.

**Figure 4:**
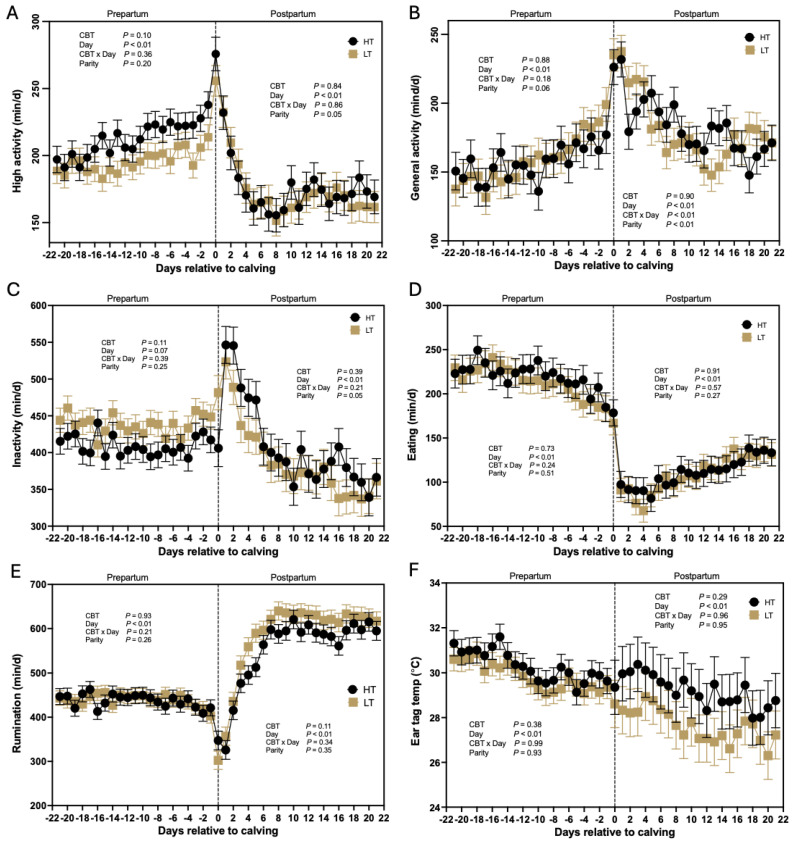
Daily minutes (least-squares means ± SEM) quantified as (**A**) high activity; (**B**) general activity; (**C**) inactivity; (**D**) eating; (**E**) rumination; and (**F**) average daily ear-surface temperature”. All measurements were collected from d −21 through +21 (d 0 = calving) for cows classified either as high-temperature (HT; black line) or low-temperature (LT; gold line). Data collected during the prepartum and postpartum periods were analyzed separately. Core body temperature (CBT) = HT vs. LT groups of cows.

Behavior data from the visual observations (e.g., lying, standing, eating, drinking, and perching; Table 2) were collected and analyzed separately for the far-off and close-up periods. Similarly, behavioral data collected using the accelerometer (e.g., periods of high activity, general activity, inactivity, eating, rumination, and ear surface temperature) were analyzed separately for the prepartum and postpartum periods. For example, prepartum rumination was analyzed independently from postpartum rumination. The percentage of time attributed to each behavior (visual observations; Table 2) or the designated daily minutes assigned to each behavior by the accelerometer (Figure 4A–F) were analyzed by fitting mixed-effect models using the MIXED procedure of SAS. All models included the fixed effects of CBT, parity (primiparous vs. multiparous), time, and the two-way interactions of CBT and parity, and CBT and time. Additional covariates offered to all models included gestation of twins (yes or no), and the average, minimal, and maximum daily ambient THI for the far-off and close-up pens, whenever available (e.g., THI data were not collected for the postpartum period). Covariates were removed from the models using a stepwise backward elimination method when *p* > 0.10. The covariance structure for the repeated measures within subjects was selected based on model convergence and fit statistics assessed using the smallest Akaike’s Information Criteria. The random effect of replicate nested within CBT was considered in all models. The lsmeans statement was employed to compute the least-squares means (LSMEANS) for factors or interactions of interest and utilized the pdiff option with Tukey’s adjustment to compute post hoc pairwise differences between LSMEANS adjusted for multiple comparisons. Statistical significance was defined as *p* < 0.05 and tendencies as 0.05 < *p* ≤ 0.15.

### 2.8. Analysis of Gene Expression in White Blood Cells

The transcriptome of circulating WBCs was evaluated in a subgroup of HT (*n* = 5) and LT (*n* = 5) cows following 7 days of temperature data collection. BGI Genomics (Beijing, China) performed RNA library preparation and sequencing. Raw sequence reads (fastq) were processed using fqtrim (https://ccb.jhu.edu/software/fqtrim/ accessed on 22 February 2024) to remove sequence adaptors and perform quality trimming. The read quality was examined using FastQC (Babraham Bioinformatics, Cambridge, UK). Trimmed paired-end reads were aligned to the Ensembl (https://useast.ensembl.org/ accessed on 22 February 2024) *Bos taurus* reference genome (ARS-UCD1.3) using Hisat2 [32]; https://daehwankimlab.github.io/hisat2/ accessed on 22 February 2024. FeatureCounts was employed to quantify the number of reads mapped to exon sequences of annotated genes using the *Bos taurus* (ARS-UCD1.3) gtf annotation file from Ensembl [33]. The differentially expressed genes (DEGs) were calculated from the read counts using a generalized linear model on edgeR robust [34].

## 3. Results

### 3.1. CBT Classification

Median CBT values for each of the five replicates were as follows: 38.78 °C, 38.85 °C, 38.84 °C, 38.92 °C, and 38.67 °C for groups 1 through 5, respectively. Core body temperature ranged from 37.4 to 41.2 °C for HT cows and from 37.4 °C to 40.5 °C for LT cows. Core body temperature was greater (*p* < 0.01) for HT cows than LT cows (Figure 3; Table 1). The average CBT of HT and LT cows varied throughout the day, spanning morning to evening, as illustrated in Figure 3. Considering all cows, CBT dropped from midnight to around 9 a.m., then rose sharply around noon, and continued to increase during the afternoon, peaking at around 6 p.m., before beginning to decline again around 8 p.m. (Figure 3).

### 3.2. Temperature–Humidity Index in the Far-Off and Close-Up Pens and Overall Descriptive Data

During the study, the overall THI daily average (Average THI), average minimum (Min THI), and average maximum (Max THI) for the far-off pen were 75.9, 60.8, and 85.7, respectively. Daily average THI, Min THI, and Max THI for the far-off pen are depicted in Figure 2A. In the close-up pen, the overall average THI, Min THI, and Max THI were 69.2, 50.3, and 82.6, respectively (Figure 2B). The observed THI during the far-off and close-up periods suggests conditions consistent with heat stress [3,4,5].

Lactation number at enrollment was greater (*p* = 0.05) for HT compared to LT cows, and the percentage of multiparous cows tended to differ (*p* = 0.15) between groups. However, there was no effect of parity on CBT (*p* = 0.23). Days in milk at dry-off did not differ (*p* = 0.94) between HT and LT cows (Table 1). Days of gestation at enrollment were lower (*p* = 0.04) for LT cows compared with HT cows. Cows classified as HT spent fewer days in the close-up pen (*p* = 0.03) compared with LT cows (Table 1). Gestation length tended (*p* = 0.09) to be shorter in HT than LT cows (Table 1). Body condition scores at enrollment (*p* = 0.94), and on days 7 (*p* = 0.78) and 14 (*p* = 0.36) post enrolment were not associated with CBT. Furthermore, twin gestation was not associated with CBT (*p* = 0.28); two HT cows and one LT cow delivered twins. Projected 305 d mature equivalent milk yield for the subsequent lactation did not differ (*p* = 0.90) between HT and LT cows.

### 3.3. Visual Behavior Monitoring

#### Far-Off Period

For the visual observations (Table 2), there were no effects of CBT (*p* ≥ 0.18) on lying, standing, eating, drinking, or perching behaviors while cows were in the far-off pen. There were, however, effects of time of day (*p* < 0.01) during which observations were performed (A.M. vs. P.M.) on lying (A.M. = 60.0% ± 3.1; P.M. = 28.1% ± 3.1), standing (A.M. = 19.2% ± 2.6; P.M. = 35.1% ± 2.6), eating (A.M. = 8.7% ± 1.8; P.M. = 25.3% ± 1.8), and drinking (A.M. = 1.6% ± 0.4; P.M. = 2.4% ± 0.4) behaviors. Furthermore, the interaction between CBT and time (*p* = 0.02) was significant for drinking behavior, as HT cows were observed more frequently drinking water in the A.M. observations (1.8% ± 0.7 vs. 1.5% ± 0.7) but less often in the P.M. observations than LT cows (1.2% ± 0.7 vs. 3.6% ± 0.7). The interaction of CBT and parity tended to be significant (*p* = 0.15) for lying behavior, as primiparous LT cows were observed more frequently lying down than primiparous HT cows (52.1% ± 4.0 vs. 41.2% ± 5.2) but no differences (*p* = 0.82) were observed between LT and HT multiparous cows. Furthermore, there were no significant effects of parity (*p* ≥ 0.21) on any of the observations during the far-off period.

### 3.4. Close-Up Period

During the close-up period, there were no effects of CBT (*p* ≥ 0.35) on lying, standing, and eating behaviors. However, there was a trend (*p* = 0.09) suggesting that HT cows drank water more frequently than LT cows during the close-up period. Furthermore, the interaction between CBT and parity affected (*p* = 0.04) drinking behavior, as HT primiparous cows were observed more frequently drinking water than LT primiparous cows (2.6% ± 0.5 vs. 2.1% ± 0.4), although no differences in drinking behavior were observed for multiparous cows based on CBT (*p* = 0.73). Furthermore, parity affected drinking behavior (*p* = 0.01), as multiparous cows (1.6% ± 0.2) were observed less frequently drinking water compared to primiparous cows (2.3% ± 0.3) during the close-up period. Additionally, the interaction between CBT and parity was associated with (*p* < 0.01) eating behavior during the close-up period, as HT multiparous cows were observed less often eating than the LT multiparous cows (12.3% ± 1.6 vs. 18.8% ± 1.8), but HT primiparous cows were observed more often eating than LT primiparous cows (21.8% ± 2.8 vs. 13.4% ± 1.9). Parity also tended (*p* = 0.15) to be associated with standing behavior, as multiparous cows were observed more frequently standing (35.4% ± 1.8 vs. 30.8% ± 2.6) than primiparous cows. Additionally, the time of day during which observations were performed (A.M. vs. P.M.) was associated (*p* < 0.01) with the percentage of time cows were observed lying (A.M. = 81.5% ± 2.1; P.M. = 15.1% ± 2.1), standing (A.M. = 11.4% ± 1.9; P.M. = 54.8% ± 1.9), eating (A.M. = 6.5% ± 1.3; P.M. = 26.6% ± 1.3), and drinking (A.M. = 0.6% ± 0.3; P.M. = 3.3% ± 0.3).

### 3.5. Automated Behavior Monitoring

#### 3.5.1. Periods of High Activity

Based on data from the AMS, HT cows exhibited a trend (*p* = 0.10) towards longer periods of high activity compared to LT cows (212 ± 7 vs. 195 ± 7 min) during the prepartum period (Figure 4A). Additionally, days relative to calving significantly influenced (*p* < 0.01) the occurrence of heightened activity behavior during the prepartum period, with a notably increase in high activity minutes around the time of parturition (day 0; Figure 4A). However, there was no observed interaction between CBT and day (*p* = 0.36), nor was there an effect of parity (*p* = 0.20) on the daily duration of high activity minutes during the prepartum period. In contrast, during the postpartum period (days 0–21; Figure 4A), there was no effect of CBT groups (*p* = 0.84) or an interaction between CBT and days relative to calving (*p* = 0.86) on daily minutes of high activity. However, there was an effect of day (*p* < 0.01), as daily high-activity minutes dropped significantly following calving (Figure 4A). Parity also affected (*p* = 0.05) the daily high-activity minutes during the postpartum, as primiparous cows (190 ± 10 min) were more active than multiparous cows (165 ± 7 min), but no significant interaction was observed between parity and CBT (*p* = 0.58).

#### 3.5.2. General Activity

Prepartum general activity (Figure 4B) was not different (*p* = 0.88) between HT and LT cows, with HT cows averaging 157 ± 11 and LT cows 159 ± 10 min of general activity, respectively. In contrast, days relative to calving affected (*p* < 0.01) the duration of general activity minutes, with a marked increase in daily active minutes at time of calving (Figure 4B). Furthermore, parity tended (*p* = 0.06) to influence daily overall prepartum activity, as there was a trend for higher general activity in multiparous (172 ± 9 min) compared to primiparous (144 ± 12 min) cows. However, there was no interaction of parity and CBT groups (*p* = 0.30), or between CBT and days relative to calving (*p* = 0.18; Figure 4B). Similarly, during the postpartum period, general activity was not different (*p* = 0.90) between HT (183 ± 8 min) and LT (181 ± 7 min) cows. However, there was an effect of parity (*p* < 0.01), as multiparous cows had greater daily activity than primiparous cows (198 ± 7 min vs. 167 ± 9 min, respectively). Nonetheless, the interaction of CBT and parity was not significant (*p* = 0.73). Furthermore, days relative to calving affected postpartum activity, as it decreased (*p* < 0.01) from a peak at parturition, through d 21. The interaction of CBT and day was also significant (*p* < 0.01), as minutes of general activity in the postpartum differed between HT and LT cows for select days (Figure 4B).

#### 3.5.3. Inactive Daily Minutes

Cows classified as HT tended (*p* = 0.11) to spend less time inactive than LT cows during the prepartum period (410 ± 13 vs. 438 ± 12 min; Figure 4C). Days relative to calving tended (*p* = 0.07) to be associated with inactive daily minutes, but there was no interaction between CBT and day (*p* = 0.39), nor was there an effect of parity (*p* = 0.25). From a peak following calving, inactive daily minutes slowly decreased (*p* < 0.01) until d 21 postpartum (Figure 4C). Core body temperature did not affect (*p* = 0.39) postpartum inactivity, with HT cows spending an average of 409 ± 16 min per day being inactive compared with LT cows at 391 ± 14 min per day (Figure 4C). There was, however, an effect of parity (*p* = 0.05), as multiparous cows spent more time inactive (420 ± 12 min) than primiparous cows (380 ± 17 min). Nonetheless, the interaction of CBT and parity was not significant (*p* = 0.41).

#### 3.5.4. Time Eating

The average time cows spent eating daily during the prepartum period was not affected by CBT (*p* = 0.73), averaging (220 ± 14 vs. 214 ± 12 min) for HT and LT cows, respectively (Figure 4D). There was a significant effect of days relative to calving (*p* < 0.01) on daily time spent eating, with a notable decrease as calving approached. In contrast, postpartum eating time was at a nadir around the time of calving, and slowly increased (*p* < 0.01) to d 21 (Figure 4D). Postpartum eating time was not affected by CBT (*p* = 0.91) and averaged 114 ± 13 min for HT cows and 112 ± 11 min for LT cows. Furthermore, there was no interaction between CBT and days relative to calving (*p* = 0.57), nor was there an effect of parity (*p* = 0.27).

#### 3.5.5. Rumination Time

Core body temperature did not have a significant effect (*p* = 0.93) on the duration of time cows spent ruminating before calving, with HT cows averaging 437 ± 15 min and LT cows averaging 439 ± 15 min (Figure 4E). Rumination time prepartum decreased as parturition approached (*p* < 0.01), and rapidly increased postpartum (*p* < 0.01) until reaching a peak at the end of the first week. Importantly, there was a tendency for the effect of CBT (*p* = 0.11) on postpartum rumination, (Figure 4E) indicating that HT cows spend less time ruminating compared with LT cows after calving (549 ± 14 vs. 580 ± 14 min). No differences (*p* = 0.35) in postpartum rumination time were observed between primiparous and multiparous cows (Figure 4E).

#### 3.5.6. Ear Surface Temperature

Although CBT was not significantly associated (*p* > 0.29) with ear surface temperature prepartum or postpartum, a trend can be seen for lower ear-surface temperatures in LT than HT throughout the peripartum period (d −21 to 2; Figure 4F). Days relative to calving had an effect (*p* < 0.01) on ear surface temperature, because temperature decreased in the prepartum as cows approached parturition. Parity (*p* ≥ 0.93) and the interaction of CBT and days relative to calving (*p* ≥ 0.96) did not affect ear surface temperature during the pre- and postpartum periods.

### 3.6. Gene Expression Differences in WBC from HT and LT Cows

**Figure 5:**
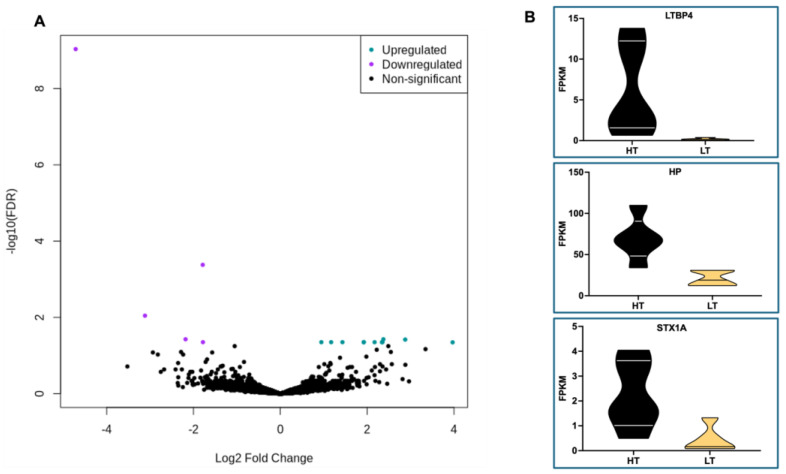
Volcano plot (**A**) highlighting the 16 differently expressed genes (DEG; FDR < 0.05; Table 3) in white blood cells (WBCs) from Holstein dry cows classified as either high-median (HT) or low-median (LT) core body temperature (CBT). Purple dots represent DEGs (*n* = 5) that were downregulated in WBCs of LT compared to HT cows, and turquoise dots represent the upregulated genes (*n* = 11) in WBCs of LT compared to HT cows. Violin plots (**B**) illustrate expression levels of the top three (*LTBP4*, *HP*, and *STX1A*) upregulated genes in WBCs of HT compared to LT cows.

RNA sequencing analysis revealed only 16 DEG (FDR < 0.05) in WBC of HT compared to LT cows (Table 3 and Figure 5A). The expression of 5 genes increased, while 11 genes decreased in WBCs of HT compared to LT cows, respectively. Furthermore, 5 out of 16 DEGs were uncharacterized (Table 3). The limited number of characterized DEGs posed a constraint on the feasibility of conducting meaningful functional analyses. Therefore, the DEGs were explored individually.

The expression levels of the top three DEGs are depicted in violin plots in Figure 5B, and increased in WBCs from HT compared to LT cows (Figure 5A, Table 3). The *LTBP4* gene, or latent transforming growth factor beta binding protein 4, encodes a binding protein for transforming growth factor beta (TGFB) [35], an important cytokine involved in both pro- and anti-inflammatory responses [36]. The *HP* gene encodes haptoglobin, a major acute-phase protein secreted primarily by the liver and lungs [37]. The *STX1A* gene encodes the syntaxin 1A protein, part of the SNARE (Soluble NSF Attachment Receptor) complex [38], that mediates the fusion of vesicles with targeted membranes, playing a major role in calcium-dependent exocytosis and endocytosis. Furthermore, the fourth (ENSBTAG00000058633) and fifth (ENSBTAG00000034366) DEGs which increased in WBCs of HT compared to LT cows are classified as novel protein-coding genes by Ensembl. ENSBTAG00000058633 encodes a protein comprising 372 amino acids, aligning with the UniProt (https://www.uniprot.org/ accessed on 4 March 2024) identifier (A0A3Q1LIM9), denoting an immunoglobulin-like domain-containing protein. ENSBTAG00000034366 encodes a protein comprising 211 amino acids, aligning with the UniProt identifier (F1N3V3), encoding the regulator of g-protein signaling 2 (RGS2). Members of the RGS family are regulatory proteins capable of deactivating the G protein subunits Gi alpha, Go alpha, and Gq alpha subtypes [39].

There were 11 genes with increased expression in WBCs of LT compared to HT cows (Figure 5A). The upregulated genes in WBCs of LT cows encode proteins associated with mRNA processing and export from nucleus (*THOC7*) [40], cell signaling (*CNKSR2, TGFBR3, ADGRL1, RIMS3*) [41,42,43,44], immunoglobulin synthesis (ENSBTAG00000054086, ENSBTAG00000048423), cell cycle regulation (*CDCA7*) [45], solute carrier transporters (*SLC9B2, SLC29A4*), and, lastly, a paternally expressed imprinted gene, the retrotransposon-derived protein *PEG10*, with known roles in cell proliferation, differentiation, and apoptosis [46].

## 4. Discussion

To our knowledge, this is the first study demonstrating a relationship between CBT and activity during the prepartum period in Holstein cows amidst the challenges of summer heat stress. We hypothesized that cows with distinct CBTs would exhibit disparate behaviors associated with different degrees of heat generation or dissipation. To monitor behavior, we employed a dual methodology incorporating visual observations and accelerometer technology. Additionally, we examined the transcriptome of WBCs of cows with distinct CBTs to potentially uncover insights into the genetic and molecular mechanisms that underlie the physiological differences in temperature regulation between HT and LT cows, rendering them more susceptible or resilient to heat.

Cattle adhere to distinct time allocations for engaging in essential behaviors, including feeding, drinking, resting, socializing, and ruminating. For instance, lactating dairy cows housed in a commercial free-stall barn have been reported to spend approximately 12 h resting in stalls, 2.5 h standing in the alley, 4.3 h eating, and 2.7 h per day moving to and from the milking parlor [47]. Furthermore, dairy cows typically ruminate about 7 h per day during the transition period [48], and rumination time is positively correlated with milk yield [49]. Heat stress, however, can induce behavioral changes, such as alterations in lying duration [22,23,50], feeding behavior [50,51], and rumination time [51]. Allen et al. [22] demonstrated that standing-bout duration increases and lying-bout duration decreases as CBT rises. The same study reported that a CBT of 38.93 °C indicated a 50% likelihood that a cow would be standing [22]. Other groups [23,50] have confirmed that, as THI rises and CBT increases, standing-bout duration increases while lying-bout duration decreases. Reductions in lying time, however, have strong detrimental effects on milk production [52,53,54] and may indirectly reduce rumination time, as cows spend the majority of their time ruminating while lying down [55]. Lying duration during the postpartum period has also been associated with health outcomes in dairy cattle. For instance, Piñeiro et al. [56] reported a quadratic relationship between lying time and postpartum blood concentrations of non-esterified fatty acids (NEFAs), as cows with reduced (less than 9 h/day) or increased (greater than 15 h/day) lying time during the first two weeks postpartum had the highest NEFA concentration, at 14 ± 3 and 7 ± 3 days postpartum. Furthermore, the same study reported a linear association between lying duration and risk of ketosis (e.g., defined as β-hydroxybutyrate (BHB) ≥ 1.2 mmol/L) within the first 14 days postpartum [56]. In the present study, however, standing and lying behaviors, evaluated by visual observations during the far-off and close-up periods (Table 2) were not associated with CBT. In contrast, the interaction of CBT and parity during the far-off period tended to affect lying behavior, as primiparous HT cows were observed less frequently lying down than primiparous LT cows, although no differences were observed among multiparous cows. Our findings for the primiparous cows, therefore, reinforce the notion of reduced lying time in cows with greater CBT. Given that primiparous and multiparous cows may exhibit varying social, feeding, and lying behaviors [57], and that CBT in response to heat stress may be influenced by parity [58], the fixed effect of parity and the two-way interaction of CBT and parity were considered in all models. Furthermore, lying duration and rumination time are associated with dry matter intake (DMI), which influences heat production and CBT. West [24] reported that DMI decreased by 0.82 kg for each degree (°C) increase in average air temperature, and every kilogram of DMI equates to approximately 1.4 to 1.9 kg of milk production [59]. In the present study, eating behavior was not associated with CBT. However, the interaction between CBT and parity influenced eating behavior, as HT multiparous cows were observed less often eating than the LT multiparous, but primiparous HT cows were observed more often eating than LT primiparous cows. Despite the observed interaction with parity, other groups have reported that heat stress diminishes eating behavior [50,51]. Furthermore, cooling heat-stressed dry cows can increase DMI [60], and greater DMI during the dry period is associated with improved postpartum health outcomes in dairy cows [61,62]. While hyperthermia is observed to suppress DMI, water consumption has been reported to increase during periods of heat stress to aid in thermoregulation, particularly in high-producing dairy cows [25]. In the present study, drinking behavior during the close-up period tended to increase in HT compared to LT cows. Furthermore, the interaction between CBT and time was significant for drinking behavior during the far-off period, as HT cows were observed more frequently drinking water in the A.M. observations but less often in the P.M. observations than LT cows. In the summer months, daily ambient temperatures are at their highest during the afternoons (Figure 3), so the observed tendency suggesting that LT cows drink more water during the P.M. observations may be associated with their improved ability to maintain a lower CBT, compared to HT cows. Collectively, the lack of strong statistical differences in the behavioral metrics evaluated by visual observations in the present study may be attributed to the short time cows were observed during the prepartum (16 h, including 8 h in the far-off pen and 8 h in the close-up pen) in addition to the small sample size (*n* = 48) evaluated in the study.

Despite the limited sample size, the present study detected significant trends based on the 24 h automated sensor monitoring system employed, supporting the hypotheses of differences in behavior of dry cows with distinct CBTs. Of note, HT cows displayed higher periods of high activity and lower periods of inactivity prepartum and diminished rumination time postpartum than LT cows. The observed differences in activity likely represent an important behavioral distinction influencing CBT (e.g., heightened activity may correlate with increased heat generation). Furthermore, the trend for reduction in rumination time suggests that HT cows face health challenges following parturition. Indeed, we have previously demonstrated that dry cows classified as HT were more likely to be diagnosed with postpartum diseases than LT cows, following parturition [12]. These findings support the notion that the consequences of heat stress during the dry period extend into the postpartum period, leading to a higher incidence of postpartum disorders [8].

Limited data are available regarding the use of ear surface temperature as a predictor of cow health. Stevenson et al. [10] demonstrated that ear surface temperatures, captured by the CowManager ear tags, were highly correlated with environmental conditions (r = 0.96). Furthermore, the same publication [10] reported that ear surface temperature was not different between healthy cows and cows that developed postpartum diseases. Although CBT was not significantly associated with ear surface temperature in the present study, a trend can be seen for lower ear-surface temperatures in LT than HT during both the prepartum and postpartum periods (Figure 4F). Further research, with an increased number of animals, is encouraged to evaluate whether ear surface temperature is a useful indicator of CBT and health traits in dairy cows. However, AMSs that capture heavy breathing behavior may provide a more accurate measure of heat stress exposure than those that assess ear surface temperature [63].

The comparison of the transcriptomes of WBCs from HT and LT cows revealed that 16 genes were differently expressed (FDR < 0.05; Table 3 and Figure 5A). The small number of DEGs observed is likely due to measuring only a single time point (blood sample collected at the time of iButton removal) from five cows per CBT group (HT and LT). Future studies should consider longitudinal sampling, especially at times of day when animals experience greater heat stress. In addition, a greater number of biological replicates per CBT group should be considered. Despite these limitations, the results from the present study remain informative. For instance, the top DEG (*LTBP4*) encodes a binding protein for TGFB. Interestingly, *LTBP4* is a direct target of heat-shock protein 1 (HSP1), a major transcriptional regulator of the heat-shock response (HSR) in eukaryotic cells [64]. As a consequence of the high temperatures, HSR is induced to preserve cellular proteostasis [65]. In the current study, the expression of *LTBP4* was higher in WBCs of HT than LT cows, supporting the hypothesis of greater heat stress response in HT cows. The second highest DEG, haptoglobin (*HP*), encodes an important biomarker of inflammation and disease in dairy cattle [66]. For instance, plasma concentrations of HP protein are increased in cows with metritis, compared with healthy cows [67]. Haptoglobin expression is induced by pro-inflammatory cytokines [68] and is primarily secreted by the liver [69], although HP mRNA has been detected in several tissues [37]. Notably, plasma haptoglobin concentrations have been observed to increase in dairy cows during high ambient temperatures [70], and, in the present study, *HP* mRNA was increased in WBCs of HT compared to LT cows, likely a reflection of greater systemic inflammation in HT cows. This is an interesting finding, as we have previously shown that HT cows are more likely to develop uterine disorders (retained fetal membranes or metritis) than LT cows [9]. The third-highest DEG detected in the present study, whose expression increased in WBCs of HT compared to LT cows was the *STX1A* gene, which encodes a protein part of the SNARE complex [38]. Similar to our findings that HT cows experience greater metabolic challenges, *STX1A* has been proposed as a potential biomarker of clinical ketosis in Holstein cows, based on an RNA-seq experiment performed in whole blood [71]. The last characterized gene that increased in WBCs of HT compared to LT cows, *RGS2*, is also a direct target of the heat-shock protein 1 (HSP1). The promoter of the RGS2 gene has a binding site for HSF1, and the expression of RGS2 in smooth muscle cells from the rat aorta has been demonstrated to increase with exposure to febrile temperatures [72]. Among the genes whose expression increased in WBCs of LT compared to HT cows (*n* = 11), two genes (*THOC7* and *PEG10*) have been previously implicated with heat stress. The *THOC7* gene, or THO Complex Subunit 7, encodes a protein part of the THO complex (consisting of THO2, HPR1, THOC5, THOC6, and THOC7) involved in mRNA export from the nucleus. Notably, the export of heat-shock mRNAs during heat stress is entirely reliant on the function of the THO complex in *Drosophila melanogaster* [73] and yeast [74,75,76]. Thus, without a functional THO complex, *D. melanogaster* and yeast have a reduced ability to cope with heat stress. Therefore, the increased expression of *THOC7* in the WBC of LT cows is likely implicated in their improved capacity to tolerate heat stress. Lastly, the paternally imprinted *PEG10* gene has also been implicated in heat tolerance under heat stress in cattle [77]. To investigate the effect of cross-breeding on tolerance to heat stress, Zhang et al. [77] investigated the inheritance patterns of leukocyte transcriptome in F1 hybrid cattle (Angus males × Droughtmaster females) and their parents Red Angus (AN; *B. taurus*) and Droughtmaster (DR; *B. indicus* and *B. taurus* genetics), under heat stress. The physiological responses to heat stress in F1 hybrids were comparable to those in AN. Inheritance pattern analysis from the gene expression data partly explained the response to heat stress in the F1 hybrids, and the *PEG10* gene was identified as a key player gene involved with the paternal dominant gene expression in the hybrids. The identification of a candidate paternally imprinted gene associated with heat stress tolerance is exciting, as it would enable farmers to rapidly disseminate this beneficial trait across a larger population through artificial insemination and targeted sire selection.

## 5. Conclusions

The current study identified disparate behavioral patterns in dry Holstein cows with distinct body temperatures during the summer months, a season known for inducing heat stress in dairy cattle. Despite the limited sample size and the lack of strong statistical significance, notable trends emerged from both visual assessment and automated behavioral monitoring. These findings provided valuable insights into the reasons for the observed variations in CBT among dry cows experiencing periods of heat stress. Given that the AMS data represent continuous monitoring over 1008 h (24 h per day over 42 days) compared to only 16 h of visual assessment, our conclusions of the behavior assessment are primarily drawn from the AMS data. Importantly, HT cows tended to be more active prepartum and had decreased rumination postpartum than LT cows, indicating that HT cows, who perhaps cannot regulate their temperature as efficiently under heat stress during the prepartum period, face health challenges postpartum. Our study also identified DEGs in WBCs of cows presenting distinct body temperatures. These differences in WBC gene signature may stem from genetic predispositions associated either with the phenotype (e.g., tolerance or susceptibility to heat stress) or with distinct physiological responses resulting from the observed behavioral differences influencing heat production or dissipation. Collectively, given that rumination time is a critical indicator of postpartum health, CBT and prepartum activity patterns may serve as valuable predictors for identifying dairy cows at risk of postpartum health disorders.

## Figures and Tables

**Table 1:** Prepartum descriptive data (mean ± SEM) of cows classified as having low (LT) or high (HT) core body temperature before calving ^1^.

	Core Body Temperature (CBT) ^1^		
Item	LT	HT	*p*-Value
Number of cows	25	25	
Percentage of multiparous cows	52.0	72.0	0.15
Lactation number at enrollment	1.7 ± 0.1	2.2 ± 0.1	0.05
Average core body temperature, °C	38.70 ± 0.03	38.94 ± 0.03	<0.01
Core body temperature by parity			0.23
Primiparous, °C	38.70 ± 0.04	38.92 ± 0.05	
Multiparous, °C	38.70 ± 0.04	38.94 ± 0.03	
Days in milk at dry-off	316.8 ± 9.2	315.8 ± 9.2	0.94
Days of gestation at enrollment	225.8 ± 1.1	229.0 ± 1.1	0.04
Days spent in close-up pen	26.7 ± 1.1	23.8 ± 1.1	0.05
Gestation length, days	280.1 ± 0.8	278.0 ± 0.8	0.09

^1^ Core body-temperature group (CBT): LT = cows with vaginal temperature below the median value within replicate; HT = cows with vaginal temperature above the median value within replicate. Median values were calculated separately for each of 5 replicates based on core body-temperature data collected during 7 d between d 225 and 239 of gestation.

**Table 2:** Least-squares means ± SEM of percentage of daily time spent in each activity during the 2 h visual observations in the far-off and close-up pens.

	Core Body Temperature ^1^		*p*-Value				
Item	Low Temperature (LT)	High Temperature (HT)	CBT	Time ^2^	CBT × Time	Parity	CBT × Parity
Far-off							
Lying, %	46.5 ± 2.8	41.6 ± 3.1	0.24	<0.01	0.48	0.21	0.15
Standing, %	26.7 ± 2.7	27.6 ± 3.1	0.85	<0.01	0.39	0.81	0.81
Eating, %	15.3 ± 1.6	18.6 ± 1.8	0.18	<0.01	0.96	0.69	0.96
Drinking, % ^3^	2.24 ± 0.4	1.8 ± 0.5	0.18	<0.01	0.02	0.67	0.91
Perching, % ^3^	10.1 ± 1.6	11.5 ± 1.8	0.66	0.37	0.67	0.85	0.59
Close-up							
Lying, %	47.2 ± 2.1	49.5 ± 2.5	0.49	<0.01	0.31	0.84	0.21
Standing, %	36.2 ± 2.4	29.7 ± 2.7	0.35	<0.01	0.34	0.15	0.37
Eating, %	16.1 ± 1.3	17.0 ± 1.6	0.66	<0.01	0.61	0.32	<0.01
Drinking, % ^3^	1.8 ± 0.3	2.1 ± 0.3	0.09	<0.01	0.50	0.01	0.04

Cows were observed for a total of two A.M. sessions and two P.M. sessions in the far-off and close-up pens. Cows were in the far-off pen between d 220 and 261 of gestation and in the close-up pen from d 247 to 285 of gestation. ^1^ Core body-temperature group (CBT): LT = cows with vaginal temperature below the median value within replicate; HT = cows with vaginal temperature above the median value within replicate. Median values were calculated separately for each of 5 replicates based on core body-temperature data collected during 7 d between d 225 and 239 of gestation. ^2^ AM observations occurred between 0600 and 0800 h and P.M. observations occurred between 1600 and 1800 h. ^3^ Data were log-transformed for analysis. The mean ± SEM from the original data (not log-transformed) are presented in the table.

**Table 3:** List of differently expressed genes (DEGs) in white blood cells (WBCs) of cows classified as having low (LT) or high (HT) core body temperature before calving ^1^.

Stable IDs ^2^	Gene Name	Gene Description	FPKM (HT)^3^	FPKM (LT) ^3^	*p*-Value	FDR
Increased in HT						
ENSBTAG00000004757	*LTBP4*	Latent transforming growth factor beta binding protein 4	6.1 ± 2.6	0.2 ± 0.05	6.41 × 10^−14^	9.23 × 10^−10^
ENSBTAG00000006354	*HP*	Haptoglobin	69.0 ± 12.1	20.9 ± 4.1	5.83 × 10^−8^	4.00 × 10^−4^
ENSBTAG00000017075	*STX1A*	Syntaxin 1A	2.2 ± 0.6	0.43 ± 0.24	1.88 × 10^−6^	9.00 × 10^−3^
ENSBTAG00000058633 ^4^	-	Immunoglobulin-like domain-containing protein ^5^	10.8 ± 3.2	4.3 ± 2.3	1.30 × 10^−5^	3.80 × 10^−2^
ENSBTAG00000034366 ^4^	*RGS2* ^5^	Regulator of g-protein signaling 2 ^5^	24.6 ± 7.0	7.1 ± 1.8	3.19 × 10^−5^	4.50 × 10^−2^
Increased in LT						
ENSBTAG00000009110 ^4^	*THOC7* ^6^	THO complex subunit 7 homolog ^6^	0.6 ± 0.1	3.5 ± 1.0	1.27 × 10^−5^	3.80 × 10^−2^
ENSBTAG00000044202	*CNKSR2*	Connector enhancer of kinase suppressor of Ras 2	0.02 ± 0.02	0.09 ± 0.02	1.59 × 10^−5^	3.80 × 10^−2^
ENSBTAG00000054086 ^4^	-	Immunoglobulin V-set domain-containing protein ^5^	6.9 ± 1.6	34.4 ± 11.6	4.06 × 10^−5^	4.50 × 10^−2^
ENSBTAG00000003458	*CDCA7*	Cell division cycle associated 7	17.8 ± 2.6	34.5 ± 3.0	3.60 × 10^−5^	4.50 × 10^−2^
ENSBTAG00000040088	*SLC9B2*	solute carrier family 9 member B2	4.9 ± 2.0	13.3 ± 2.6	3.54 × 10^−5^	4.50 × 10^−2^
ENSBTAG00000048423 ^4^	-	Ig-like domain-containing protein ^5^	4.8 ± 2.3	12.12 ± 1.9	4.66 × 10^−5^	4.50 × 10^−2^
ENSBTAG00000024269	*TGFBR3*	Transforming growth factor beta receptor 3	2.7 ± 0.5	6.1 ± 0.8	4.43 × 10^−5^	4.50 × 10^−2^
ENSBTAG00000003675	*ADGRL1*	Adhesion G protein-coupled receptor L1	2.5 ± 0.7	5.2 ± 0.3	2.64 × 10^−5^	4.50 × 10^−2^
ENSBTAG00000059156	*PEG10*	Paternally expressed 10	1.2 ± 0.7	3.1 ± 0.8	4.54 × 10^−5^	4.50 × 10^−2^
ENSBTAG00000018726	*RIMS3*	Regulating synaptic membrane exocytosis 3	0.3 ± 0.1	1.5 ± 0.4	3.43 × 10^−5^	4.50 × 10^−2^
ENSBTAG00000007772	*SLC29A4*	Solute carrier family 29 member 4	0.1 ± 0.1	1.3 ± 0.5	5.02 × 10^−5^	4.50 × 10^−2^

^1^ Core body-temperature group (CBT): LT = cows with vaginal temperature below the median value within replicate. HT = cows with vaginal temperature above the median value within replicate. Median values were calculated separately for each of five replicates based on core body-temperature data collected during 7 d between d 225 and 239 of gestation. ^2^ Ensembl stable IDs (https://useast.ensembl.org/ accessed on 4 March 2024). ^3^ FPKM data are presented as mean ± SEM. ^4^ Gene name and description were unavailable in the Ensembl database (uncharacterized genes). ^5^ Protein information obtained from UniProt (https://www.uniprot.org/ accessed on 4 March 2024) matching Ensembl stable IDs. ^6^ Gene information obtained from the Innate DB database (https://innatedb.com/ accessed on 4 March 2024) matching Ensembl stable IDs.

## Data Availability

The sequence files and associated metadata for all samples utilized in this study have been securely deposited in the NCBI Sequence Read Archive (SRA) repository (BioProject accession: PRJNA1119056).
